# Quantitative Proteomics of Intracellular *Campylobacter jejuni* Reveals Metabolic Reprogramming

**DOI:** 10.1371/journal.ppat.1002562

**Published:** 2012-03-08

**Authors:** Xiaoyun Liu, Beile Gao, Veronica Novik, Jorge E. Galán

**Affiliations:** Section of Microbial Pathogenesis, Yale University School of Medicine, New Haven, Connecticut, United States of America; Harvard School of Public Health, United States of America

## Abstract

*Campylobacter jejuni* is the major cause of bacterial food-borne illness in the USA and Europe. An important virulence attribute of this bacterial pathogen is its ability to enter and survive within host cells. Here we show through a quantitative proteomic analysis that upon entry into host cells, *C. jejuni* undergoes a significant metabolic downshift. Furthermore, our results indicate that intracellular *C. jejuni* reprograms its respiration, favoring the respiration of fumarate. These results explain the poor ability of *C. jejuni* obtained from infected cells to grow under standard laboratory conditions and provide the bases for the development of novel anti microbial strategies that would target relevant metabolic pathways.

## Author Summary

*Campylobacter jejuni* is one of the most common causes of food-borne illness in the United States and a major cause of diarrheal diseases in developing countries. This pathogen can invade intestinal epithelial cells, which is very important for its ability to cause disease. Once it gains access to epithelial cells, *C. jejuni* becomes unable to grow under standard growth conditions, although it can grow if pre-incubated under oxygen limiting conditions. This study compares the protein compositions of *C. jejuni* grown under standard growth conditions and obtained from within epithelial cells. This analysis indicates that, within cells, *C. jejuni* undergoes a significant metabolic downshift and reprograms its respiration, favoring the respiration of fumarate. These results may provide the bases for the development of novel anti microbial strategies that would target relevant metabolic pathways.

## Introduction

*Campylobacter jejuni* is the leading cause of bacterial food-borne disease in the USA and Europe and a major cause of diarrheal disease in developing countries [1], [2]. The typical route of *C. jejuni* transmission is through handling and ingesting contaminated food, milk, or water [3]. In many cases, poultry is a common source of food contamination, and the gastrointestinal tract of many species of birds often serves as the natural reservoir of *C. jejuni* [4]. Despite its important impact on public health, surprisingly little is known about its pathogenesis [5]. Biochemical analysis and comparative genomic studies of *C. jejuni* have revealed unique features in its metabolic pathways [6]–[8]. Unlike most enteric bacteria, *C. jejuni* lacks the key glycolytic enzyme 6-phosphofructokinase as well as alternative pathways for sugar catabolism. Consequently, *C. jejuni* utilizes amino acids and organic acids as the major carbon sources. In addition, *C. jejuni* possesses highly branched electron transport chains, which allow it to respire not only oxygen but also a variety of electron acceptors including fumarate, nitrate, nitrite, trimethylamine-N-oxide (TMAO), and dimethyl sulfoxide (DMSO) [9]–[12]. Such remarkable diversity in its respiratory pathways may contribute to its ability to survive or grow in substantially different environments [13].

We previously reported that *C. jejuni* survives within cultured intestinal mammalian cells by avoiding its delivery to lysosomes [14]. We also found that 24 hours after infection, *C. jejuni* recovered from cultured intestinal mammalian cells cannot be efficiently cultured under microaerophilic conditions unless it is pre-incubated under oxygen-limiting conditions. We hypothesized that within host cells *C. jejuni* may alter its physiology and change its mode of respiration perhaps due to low level of oxygen within the *C. jejuni*-containing vacuole. Such metabolic change should most likely be reflected in alterations on the levels of different sets of metabolic enzymes.

In an effort to understand the physiological changes in intracellular *C. jejuni* we surveyed its proteome at different time after infection of cultured mammalian cells and compared it to the proteome of bacteria grown *in vitro*. Our data indicate that within cultured mammalian cells, *C. jejuni* undergoes a severe metabolic downshift and an apparent change of its respiration mode. These data provide major insight into the *in vivo* metabolism and pathogenesis of this important pathogen.

## Results/Discussion

### Overview of the proteomic analysis of intracellular *C. jejuni*

**Figure 1:**
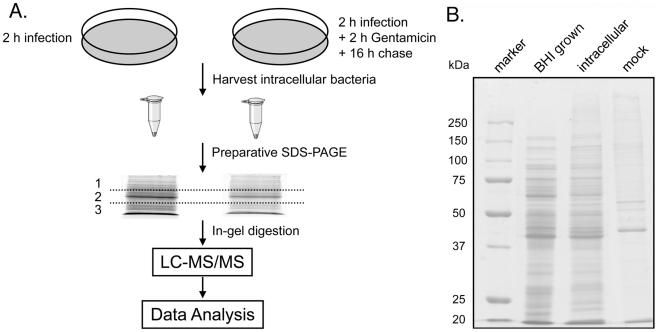
A proteomics approach to profile intracellular *C. jejuni*. **A** A schematic diagram of the experimental approach used in this study. **B** SDS-PAGE analysis of extracted proteins from *C. jejuni* samples. Proteins obtained from intracellular bacteria isolated following the protocol described above (intracellular), from equal number of bacteria obtained after growth in Brain Heart Infusion broth (BHI grown) or from uninfected cells subjected to the same protocol (mock), were separated by SDS-PAGE and stained with Coomassie brilliant blue.

To obtain a snapshot of *C. jejuni* protein expression during infection, we developed a protocol to isolate bacteria away from mammalian host cell proteins by exploiting *C. jejuni*'s moderate resistance to detergent treatment. This protocol resulted in the presence of very low amounts (<15%) of host cell-derived proteins in the final preparation. COS-1 cells were infected with *C. jejuni* and 2 hs and 20 hs after infection, intracellular bacteria were isolated, and proteins from bacterial lysates were pre-fractionated by gel electrophoresis prior to LC-MS/MS analysis. We chose to compare these two time points because while at 2 hs after infection *C. jejuni* is readily culturable under microaerophilic conditions, at 20 hs after infection it is not [14]. We also reasoned that after 2 hs of infection, the *C. jejuni* proteome would closely resemble that of extracellular bacteria allowing the opportunity to examine the potential changes that may occur during the transition from an extracellular to an intracellular niche. More importantly, the comparison of proteomes of bacteria isolated by similar procedures minimized potential differences due to sample processing that could occur by comparing the proteomes of bacteria grown in culture with that of bacteria obtained from within mammalian cells. Samples generated with the outlined protocol (see Figure 1A) exhibited limited host-protein contamination when examined by both Coommassie blue staining after SDS-PAGE (Figure 1B), and mass spectrometry analysis (less than 15% of the identified proteins corresponded to the mammalian cell proteome). Through the assignments of ∼400,000 MS/MS spectra we were able to detect 1,428 *C. jejuni* proteins, which represent ∼86% of the entire *C. jejuni* proteome (see Dataset S1). We used spectral counting to assess the relative protein abundance in the different samples [15]. Most proteins in the dataset were assigned from multiple spectral counts, which combined with the numerous technical and biological replicates, provided strong confidence to the protein assignments. In addition we quantified a selected group of proteins by selective reaction monitoring (SRM) (see below) [16]. Our data showed that proteomic differences determined by spectral counts agreed well with those measured by SRM although in some cases (i. e. proteins present in large amounts) spectral counting slightly underestimated the extent of proteomic differences (reported as the fold changes) between samples (see below). Such observation is consistent with the fact the dynamic range of spectral counting is not as high as that of SRM [16]. To our knowledge, this represents the most comprehensive *C. jejuni* proteomic dataset available.

### *C. jejuni* remodels its proteome within cultured mammalian cells

After applying the criteria described in Materials and Methods, we found 225 proteins whose levels at 20 hs after infection differed significantly from those at 2 hs after infection (Dataset S2). The vast majority of these proteins (211) showed significantly (*p*<0.05) reduced levels at 20 hs after infection. Only a few proteins showed a very moderate increase in their levels at 20 hs post-infection (Dataset S2). As predicted, the proteome of *C. jejuni* grown *in vitro* closely resembled the proteome of *C. jejuni* isolated from within cultured mammalian cells 2 hs after infection (Dataset S3). We specifically examined the profile of proteins whose levels are known to increase as a consequence of general stress responses [17]–[19] and found that their levels were either unchanged or were even slightly decreased (Table 1). Furthermore, the levels of proteins that are thought or known to protect *C. jejuni* against oxidative stress [20] were also either decreased or unaltered (Table 1), although their levels were relatively high in all time points. These results suggest that *C. jejuni* may not be subject to environmental stress when located within cultured mammalian cells and have important implications for the understanding of *C. jejuni* intracellular stage during infection.

**Table 1:** Levels of *C. jejuni* proteins associated with oxidative stress.

			Abundance^1^			
Gene ID	Gene symbol	Protein name	2 h^2^	20 h^3^	Fold^4^	p-value^5^
CJJ81176_1234	*groEL*	chaperonin GroEL	322	308	−1.06	0.351
CJJ81176_0775	*dnaK*	molecular chaperone DnaK	130	96	−1.36	<0.001
CJJ81176_1242	*htrA*	protease DO	101	81	−1.24	0.004
CJJ81176_1233	*groES*	co-chaperonin GroES	13	4	−3.13	0.002
CJJ81176_0774	*grpE*	co-chaperone protein GrpE	4	4	−1.04	0.660
CJJ81176_0537	*clpB*	ATP-dependent chaperone protein ClpB	32	19	−1.66	0.001
CJJ81176_1243	*dnaJ-1*	co-chaperone protein DnaJ	4	2	−1.64	0.125
CJJ81176_1288	*spoT*	RelA/SpoT family protein	<1	1	n.a^6^	n.a
CJJ81176_0356	*ahpC*	anti-oxidant AhpCTSA family protein	126	110	−1.15	0.017
CJJ81176_0298	*ahpC*	anti-oxidant AhpCTSA family protein	4	3	−1.31	0.277
CJJ81176_0205	*sodB*	superoxide dismutase, Fe	28	20	−1.35	0.020
CJJ81176_0800	*tpx*	thiol peroxidase	101	118	1.16	0.022
CJJ81176_0183	*trx*	thioredoxin	47	20	−2.17	0.005
CJJ81176_0291		biotin sulfoxide reductase	25	8	−3.17	<0.001
CJJ81176_1387	*katA*	catalase	11	15	1.59	0.023
CJJ81176_0182	*trxB*	thioredoxin-disulfide reductase	18	4	−4.06	0.002
CJJ81176_1519		bacterioferritin, putative	56	38	−1.53	0.004

1 Protein abundance is indicated as averaged spectral counts from 7 biological replicates.

2 *C. jejuni* samples isolated from host cells at 2 h of infection.

3 *C. jejuni* samples isolated from host cells at 20 h of infection.

4 Fold change in protein abundance; positive or negative values indicate higher or lower levels in the 20 h samples, respectively.

5 p-values were calculated using the paired Student's *t*-test.

6 Data not available (due to low protein signal).

### *C. jejuni* undergoes a metabolic downshift within cultured mammalian cells

**Figure 2:**
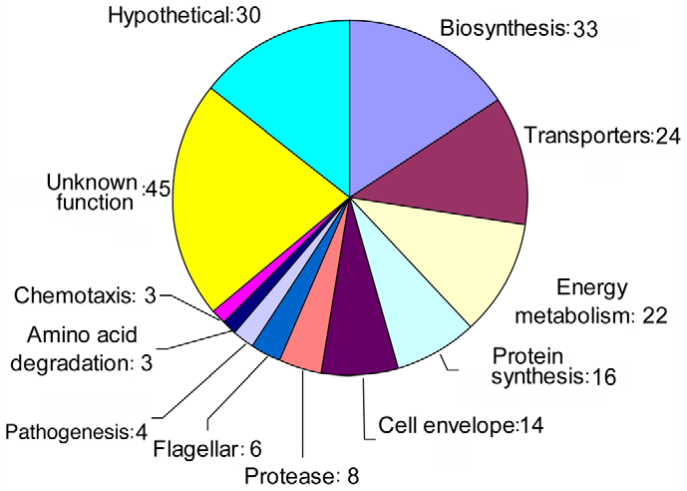
Functional categorization of *C. jejuni* proteins whose expression was lower in samples obtained 20 hours after infection. Proteins were assigned to the different functional groups based on genome annotation and additional curation based on published literature.

**Figure 3:**
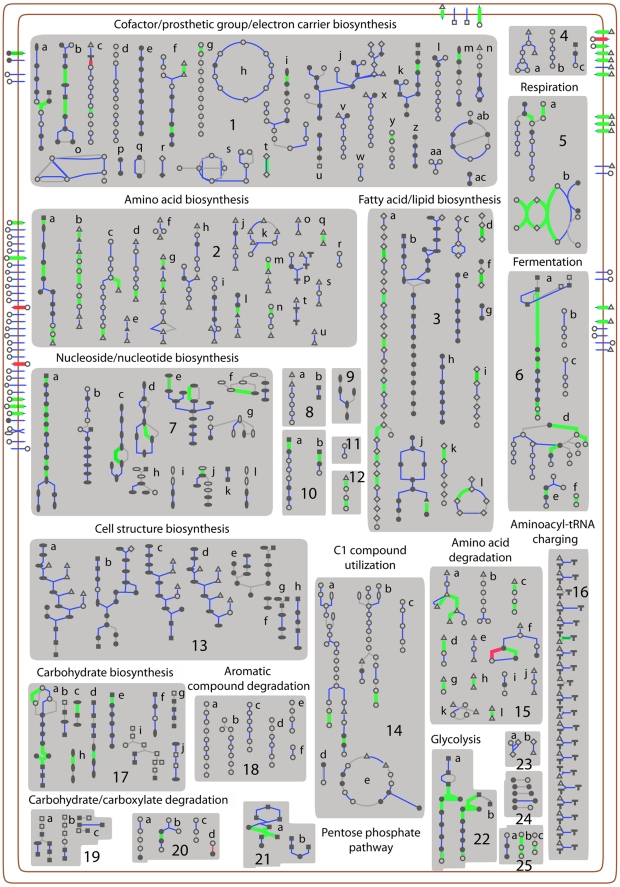
Cellular overview of *C. jejuni* metabolic pathways. Metabolic pathways are grouped into different general functional categories as indicated. Protein transporters are located at the double peripheral lines representing the bacterial membrane structure. Within individual pathways, symbols represent metabolites and connecting lines denote respective enzymes that catalyze the inter-conversion of those metabolites. Most enzymes are color-coded in the following fashion: blue indicates mapped proteins in pathways present in the *C. jejuni* proteome, grey indicates those not found in the annotated genome, bold green represents those proteins whose expression levels were lower at 20 h of infection relative to 2 h of infection, and bold red corresponds to up-regulated proteins at 20 h of infection. Description of individually numbered pathways is as follows: **1**. a, flavin biosynthesis; b, methylerythritol phosphate pathway; c, tetrapyrrole biosynthesis; d, ubiquinone-8 biosynthesis; e, di-trans, poly-cis-undecaprenyl phosphate biosynthesis; f, pantothenate biosynthesis I; g, chlorophyllide a biosynthesis I; h, TCA cycle; i, tetrahydrofolate biosynthesis; j, thiamine biosynthesis I; k, pyridoxal 5′-phosphate biosynthesis; l, coenzyme M biosynthesis; m, 6-hydroxymethyl-dihydropterin diphosphate biosynthesis; n, NAD biosynthesis I; o, folate transformations p. phosphate acquisition I; q, NAD phosphorylation and dephosphorylation; r, acyl carrier protein metabolism; s, formyl THF biosynthesis I; t, thioredoxin pathway; u, NAD salvage pathway II; v, biotin biosynthesis; w, menaquinone-8 biosynthesis; x, biotin biosynthesis; y, heme biosynthesis from uroporphyrinogen-III II; z, trans, trans-farnesyl diphosphate biosynthesis; aa, glutathionylspermidine biosynthesis; ab, NAD salvage pathway I; ac, geranyldiphosphate biosynthesis. **2**. a, histidine biosynthesis; b, lysine biosynthesis I; c, valine biosynthesis; d, isoleucine biosynthesis I; e, threonine biosynthesis from homoserine; f, homocysteine biosynthesis; g, homoserine biosynthesis; h, ornithine biosynthesis; i, tryptophan biosynthesis; j, proline biosynthesis I; k, S-adenosyl-L-methionine cycle I; l, serine biosynthesis; m, alanine biosynthesis; n, tyrosine biosynthesis I; o, alanine biosynthesis III; p, selenocysteine biosynthesis I; q, glutamate biosynthesis; r, protein citrullination; s, cysteine biosynthesis I; t, L-glutamine biosynthesis II; u, glutamine biosynthesis I; **3**. a, palmitate biosynthesis II; b, CMP-KDO biosynthesis I; c, biotin-carboxyl carrier protein assembly; d, fatty acid biosynthesis initiation III; e, CDP-diacylglycerol biosynthesis I; f, fatty acid biosynthesis initiation II; g, cyclopropane fatty acid (CFA) biosynthesis; h, CDP-diacylglycerol biosynthesis II; i, cis-vaccenate biosynthesis; j, phospholipid biosynthesis I; k, stearate biosynthesis II; l, fatty acid elongation-saturated. **4**. a, (5R)-carbapenem biosynthesis; b, hyperforin biosynthesis; c, myo-inositol biosynthesis. **5**. a, respiration (anaerobic); b, aerobic respiration-electron donor II. **6**. a. homolactic fermentation; b. (S)-acetoin biosynthesis; c. (R)-acetoin biosynthesis I d. mixed acid fermentation; e. pyruvate fermentation to acetate; f. pyruvate fermentation to lactate. **7**. a, 5-aminoimidazole ribonucleotide biosynthesis I; b, uridine-5′-phosphate biosynthesis c, adenosine nucleotides de novo biosynthesis; d, guanosine nucleotides de novo biosynthesis; e, pyrimidine deoxyribonucleotides de novo biosynthesis; f, salvage pathways of pyrimidine ribonucleotides; g, salvage pathways of guanine, xanthine, and their nucleosides; h, salvage pathways of purine and pyrimidine nucleotides; i, salvage pathways of purine and pyrimidine nucleotides; j, salvage pathways of purine and pyrimidine nucleotides; k, salvage pathways of purine and pyrimidine nucleotides; l, salvage pathways of purine and pyrimidine nucleotides. **8**. a, autoinducer AI-2 biosynthesis I; b, PRPP biosynthesis I. **9**. ppGpp biosynthesis. **10**. a, chorismate biosynthesis I; b, 3-dehydroquinate biosynthesis I. **11**. IAA biosynthesis V. **12**. putrescine biosynthesis II. **13**. a, UDP-N-acetylmuramoyl-pentapeptide biosynthesis I (generic); b, superpathway of KDO_2_-lipid A biosynthesis; c, peptidoglycan biosynthesis III; d, UDP-N-acetylmuramoyl-pentapeptide biosynthesis III (meso-DAP); e, enterobacterial common antigen biosynthesis; f, O-antigen biosynthesis; g, dTDP-L-rhamnose biosynthesis I; h, UDP-N-acetyl-D-glucosamine biosynthesis I. **14**. a, purine degradation II (anaerobic); b, purine degradation III (anaerobic); c, formaldehyde oxidation V (tetrahydrofolate pathway); d, reductive monocarboxylic acid cycle; e, formaldehyde assimilation I (serine pathway). **15**. a, aspartate-glutamate-proline degradation; b, tyrosine degradation I; c, arginine degradation IV; d, aspartate degradation II; e, proline degradation II; f, L-serine degradation; g, lysine degradation I; h, glutamine degradation II; i, citrulline degradation; j, proline degradation I; k, phenylalanine degradation (aerobic); l, asparagine degradation I. **16**. tRNA charging pathway. **17**. a, gluconeogenesis I; b, colanic acid building blocks biosynthesis; c, colanic acid building blocks biosynthesis; d, GDP-mannose biosynthesis; e, ADP-L-glycero-beta-D-manno-heptose biosynthesis; f, CMP-KDO biosynthesis II; g, GDP-glucose biosynthesis; h, GDP-D-rhamnose biosynthesis; i, glycogen degradation I; j, CMP-N-acetylneuraminate biosynthesis II. **18**. a, protocatechuate degradation I; b, methylgallate degradation; c, protocatechuate degradation III; d, orthanilate degradation; e, cyanurate degradation; f, anthranilate degradation I (aerobic). **19**. a, galactose degradation I; b, glycogen degradation II; c, glucose and glucose-1 phosphate degradation. **20**. a, glycolate and glyoxylate degradation I; b, pyruvate fermentation to acetate VII; c, glycolate and glyoxylate degradation II; d, acetate conversion to acetyl-CoA. **21**. a, pentose phosphate pathway (non-oxidative branch); b, pentose phosphate pathway (partial). **22**. a, glycolysis I b. glycolysis II. **23**. a, seed germination protein turnover; b, wound-induced proteolysis I. **24**. fatty acid and lipids degradation. **25**. a, sulfate activation for sulfonation, b, nitrate reduction III; c, nitrate reduction IV. Open triangles: Amino acids; open squares: carbohydrates; open rhomboids: proteins; vertical ovals: purines; horizontal ovals: pyrimidines; inverted triangles: cofactors; **T**:tRNAs; open circles: other; closed symbols indicate phosphorylated forms. *(Note: this data has been deposited in the BioCyc home page [* http://biocyc.org/ *] where it can be seen in an interactive fashion)*.

Functional grouping of the proteins whose expression changed upon *C. jejuni's* transition from the extracellular to the intracellular environment indicated an over representation of proteins involved in different metabolic pathways or in the transport of nutrients or compounds across the bacterial envelope (Figure 2). To better evaluate the potential physiological impact of the reduction of expression of proteins involved in metabolic pathways or transport mechanisms, we generated a graphical metabolic network database, using the Pathway Tools v14.0 [21]. For this purpose, we input the *C. jejuni* 81–176 genome sequence [22] to the program, which can predict pathways by matching the annotated enzymes of the input genome with its own comprehensive pathway/reaction database *Metacyc* [23]. We then manually curated this computation-derived *C. jejuni* pathway database based on published literature, such as biochemical studies on metabolic enzymes, transporters or regulators specifically in *Campylobacter* species. *C. jejuni* has unique metabolic capabilities presumably to adapt to different environments, so we customized the *C. jejuni* database by adding new pathways/reactions that are not present in *Metacyc*, or by linking related pathways to form super-pathways to better visualize the metabolite flow. Overall this database could account for 155 metabolic pathways, 885 associated enzymatic reactions, 75 transporters and 49 transport reactions. In this graphic representation, *C. jejuni* proteins were mapped into individual pathways, and were differentially labeled based on whether they were detected in our analysis or whether their expression level was decreased or increased. Following this template, our proteomic analysis was able to monitor 151 metabolic pathways, 382 associated enzymes, 429 enzymatic reactions, and 41 transporters (Figure 3) (The entire database will be made available in the BioCyc Database collection web site [http://biocyc.org]). Evaluation of the proteins (and associated pathways) whose expression significantly decreased 20 hs after infection indicates that *C. jejuni* undergoes a significant metabolic downshift within host cells (Dataset S2). Several proteins associated with various anabolic pathways showed significantly reduced levels in samples obtained 20 hs after infection (Dataset S2 and Figure 3). This includes components of amino acid biosynthesis pathways such as those associated with histidine, lysine, valine, leucine, isoleucine, methionine, glycine, and alanine biosynthesis. Similarly, components of other important biosynthetic pathways including those associated with the synthesis of prostetic groups and co-factors, fatty acids and lipids, pentose phosphate, as well as purine and pyrimidine nucleotides were also significantly down-regulated 20 hs after infection. In addition, the levels of several proteins associated with various catabolic pathways were significantly reduced in *C. jejuni* isolated from cells 20 hs after infection. These include pathways involved in the degradation and utilization of amino acids (e. g. proline, L-asparatate, lysine, serine and L-arginine), and C1 compounds. Pathways potentially involved in nutrient acquisition were also down-regulated, including several amino acid ABC-type transporters as well as transporters for phosphate, potassium, tungstate and molybdate. Overall, <50% of all *C. jejuni* proteins whose levels were decreased after 20 hs of infection were associated with metabolic pathways or transport reactions. Consistent with an overall metabolic downshift, the levels of several ribosomal protein subunits (e. g. 30S ribosomal proteins S6, S19, S15, and S20, and 50S ribosomal protein L9 and L24) were significantly decreased. This metabolic downshift is consistent with the observation that *C. jejuni* does not replicate within cultured mammalian cells but, rather, it seems to go into a dormant, non-culturable state [14]. In addition, the observed metabolic downshift resembles the one observed during *C. jejuni*'s transition to late stationary phase [24]. However, unlike what was observed in *C. jejuni* stationary phase transcriptional reprogramming, we did not observe increased expression of heat shock proteins (e. g. GroEL, GroES, GrpE, ClpB) or proteins associated with oxidative stress resistance (e. g. AhpC, SodB, and Tpx). This suggests that the remodeling of the *C. jejuni* proteome that occurs within cultured mammalian cells is not simply the result of its transition to a different “growth phase” but most likely, the result of its specific adaptation to the intracellular environment.

### *C. jejuni* reprograms respiration within cultured cells

The observation that intracellularly localized *C. jejuni* becomes culturable after incubation under very low oxygen conditions suggests at least two possibilities to explain its non-culturability under standard microaerophilic conditions. One possibility is that the intracellular remodeling of its proteome renders *C. jejuni* oxygen sensitive. This hypothesis would be supported by the observation that some proteins potentially involved in conferring protection against oxidative stress showed reduced levels at 20 hs after infection (Table 1). However, even though slightly reduced, the levels of these enzymes remained relatively high. Furthermore, no significant changes in the levels of proteins thought to be most important in oxidative stress protection (e. g. SodB and KatA) [25]–[27] were observed suggesting that other factors must account for *C. jejuni*'s inability to grow under microaerophilic conditions when directly obtained from cultured cells. Consistent with this hypothesis, examination of the proteome of intracellularly-localized *C. jejuni* suggests that it undergoes reprogramming of its respiration. Indeed, the levels of many proteins that are central to the main respiration pathways were significantly reduced in the 20 hs sample (Table 2), an observation confirmed by SRM experiments (Table 3). For example, key components of the aerobic respiration pathway [7] were greatly reduced. The *C. jejuni* genome only encodes two terminal oxidases, a cbb3-type cytochrome c oxidase and a CioAB-type (cyanide-insensitive oxidase) oxidase [6]. The expression levels of cbb3 type cytochrome c oxidase (CcoP and CcoO) decreased markedly 20 hs after infection (Table 2 and Table 3). The CioAB-type oxidase could be hardly detected before and after infection, which is consistent with the hypothesis that the cbb3-type cytochrome c oxidase plays a more important role in aerobic respiration [28]. This hypothesis is also supported by the observation that a *C. jejuni ccoN* mutant was unable to colonize chickens while a *cydA* mutant colonized as well as the wild type [29]. Reduced levels of the only enzyme that directly uses O_2_ as electron acceptor may make it difficult for intracellular *C. jejuni* to grow under microaerophilic conditions after its extraction from within mammalian cells. Some components of anaerobic respiration also show reduced levels in *C. jejuni* samples obtained 20 hs after infection. For example, the levels of NapA/NrfA, involved in nitrate/nitrite respiration, and TorA/TorC, which are central components of TMAO respiration, were also markedly decreased. In contrast, the levels of the fumarate reductase FrdA between the 2 hs and 20 hs time points remained constant and relatively high suggesting that fumarate may be an important electron acceptor for *C. jejuni* during its intracellular stage. Thus, our proteomics data suggest that 20 hs after infection, aerobic respiration is greatly reduced, while the anaerobic respiration pathway can still be maintained by using fumarate as alternative electron acceptors. Furthermore, these results may help to explain *C. jejuni*'s inability to grow under standard microaerophilic conditions when plated immediately after its release from mammalian cells 20 hs after infection. Although the actual oxygen concentration within the *C. jejuni*-containing vacuole is unknown, during the infection process, *C. jejuni* are exposed to a higher oxygen tension (5% CO_2_ vs. 10% CO_2_ when grown microaerophilically) at least until they reach the intracellular environment. To evaluate if this (presumably brief) change in oxygen concentration may itself result in changes in the *C. jejuni* proteome, we compared the proteome of *C. jejuni* incubated in the infection medium under 5% CO_2_ for 2 hs (without host cells) with the proteome of in-vitro grown bacteria. However, we found no significant differences in the *C. jejuni* proteome under these two conditions (Dataset S4) indicating that exposure to the higher oxygen conditions during infection cannot account for the observed changes in the intracellular population of *C. jejuni* 20 hs after infection, which are most likely the direct consequences of its adaptation into the host intracellular environment.

**Table 2:** Levels of *C. jejuni* proteins associated with respiration/electron transport chains.

			Abundance^1^					
Protein complex	Gene ID	Gene symbol	e.c.^2^	2 h^3^	20 h^4^	O_2_-limiting^5^	Fold^6^	p-value^7^
NADH dehydrogenase^8^	CJJ81176_1556	*nuoI*	7	5	4	7	−1.27	0.049
	CJJ81176_1558	*nuoG*	15	23	20	38	−1.15	0.13
	CJJ81176_1561	*nuoD*	7	5	4	5	−1.18	0.536
flavodoxin	CJJ81176_1384	*fldA*	43	52	36	20	−1.36	0.07
cytochrome c oxidase, cbb3-type	CJJ81176_1479	*ccoP*	25	25	9	19	−2.71	<0.001
	CJJ81176_1481	*ccoO*	26	25	12	19	−2.05	<0.001
ubiquinol–cytochrome c reductase	CJJ81176_1199	*petC*	44	44	14	32	−3.08	<0.001
	CJJ81176_1200	*petB*	0	<1	<1	5	n.a^9^	n.a
	CJJ81176_1201	*petA*	17	15	4	10	−3.62	<0.001
fumarate reductase	CJJ81176_0432	*frdC*	0	<1	<1	5	n.a	n.a
	CJJ81176_0433	*frdA*	82	77	62	153	−1.21	0.023
	CJJ81176_0434	*frdB*	14	11	9	28	−1.02	0.379
	CJJ81176_0463	*mfrA*	2	1	<1	63	n.a	n.a
	CJJ81176_0464	*mfrB*	0	<1	<1	37	n.a	n.a
	CJJ81176_0465	*mfrC*	0	<1	<1	24	n.a	n.a
nitrate reductase	CJJ81176_0801	*napA*	60	51	9	85	−5.47	<0.001
	CJJ81176_0804	*napB*	5	8	3	16	−2.44	<0.001
nitrite reductase	CJJ81176_1359	*nrfA*	3	5	1	46	−5.37	0.011
sulfite reductase	CJJ81176_0403	*yedY*	4	4	2	3	−1.59	0.05
TMAO reductase	CJJ81176_0291	*torA*	28	25	8	37	−3.17	<0.001
	CJJ81176_0292	*torC*	13	11	3	9	−3.17	<0.001
cytochrome c551 peroxidase	CJJ81176_0382	*ccpA-2*	13	12	1	8	−7.32	0.001
	CJJ81176_0047	*ccpA-1*	7	7	1	2	−7.45	<0.001
sulfite oxidoreductase	CJJ81176_0031	*sorA*	5	4	3	3	−1.14	0.538
gluconate dehydrogenase	CJJ81176_0438		23	23	4	0	−5.65	<0.001
	CJJ81176_0439		86	74	29	5	−2.49	<0.001
formate dehydrogenase	CJJ81176_1501	*fdhC*	0	0	0	4	n.a	n.a
	CJJ81176_1502	*fdhB*	1	2	1	13	−3.48	<0.001
	CJJ81176_1503	*fdhA*	28	28	5	46	−5.15	<0.001

1 Protein abundance is indicated as averaged spectral counts.

2 Extracellular *C. jejuni* samples obtained from *in-vitro* culture.

3 *C. jejuni* samples isolated from host cells at 2 h of infection.

4 *C. jejuni* samples isolated from host cells at 20 h of infection.

5 *C. jejuni* samples isolated from host cells at 20 h of infection and further incubated under oxygen-limiting conditions.

6 Fold change in protein abundance; positive or negative values indicate higher or lower levels in the 20 h vs 2 h samples, respectively.

7 p-values were calculated using the paired Student's *t*-test.

8 Other proteins encoded in the same operon (*nuoNMLKJHCBA*) are not listed because they were not detected or detected at very low levels. This is also the case for other proteins relevant to respiration including CcoQN, CioAB/CydAB, NapGHLD, NrfH, YedZ, DmsABCD (dimethyl sulfoxide reductase), and SorB.

9 Data not available (due to low protein signal).

**Table 3:** SRM measurements of a subset of *C. jejuni* metabolic enzymes.

				Peak intensity^2^			Spectral counts^4^		
Protein	Peptide	Transitions^1^		2 h	20 h	Fold^3^	2 h	20 h	Fold^5^
CcoP	TANENLVAK	480.4	787.5	1.25E7	5.15E6	−2.4	22	10.7	−2.1
		480.4	471.5	5.70E5	2.90E5	−2.0			
		480.4	394.4	2.40E5	9.40E4	−2.6			
NrfA	KISEELK	424.2	605.6	2.30E5	4.30E4	−5.3	1.7	0.7	−2.4
		424.2	242.3	3.20E4	6.00E3	−5.3			
		424.2	129.1	3.90E4	7.00E3	−5.6			
AspA	VADIALER	444.0	716.5	9.10E6	3.40E6	−2.7	28	18	−1.6
		444.0	488.3	6.40E6	2.40E6	−2.7			
		444.0	417.3	3.00E6	1.10E6	−2.7			
CcpA-2	NSGLVALPK	449.9	441.0	8.90E5	N.D.^6^	N.D.^7^	8.3	2	−4.2
		449.9	697.5	3.60E5	N.D.	N.D.			
		449.9	527.3	2.80E5	N.D.	N.D.			
PetC	VGLTEAAQAK	494.5	485.5	5.00E6	3.00E5	−16.7	32.3	13.3	−2.4
		494.5	718.5	3.90E6	2.50E5	−15.6			
		494.5	617.4	2.40E6	1.50E5	−16.0			
FdhA	FGGGVNILR	467.1	515.3	2.20E5	4.90E4	−4.5	13.3	4.3	−3.1
		467.1	728.5	1.60E5	3.90E4	−4.1			
		467.1	418.3	1.00E5	2.30E4	−4.3			
FrdA	SLVDKEGK	438.8	675.4	3.50E5	4.60E5	1.3	41	42.3	1.0
		438.8	576.3	1.40E5	1.90E5	1.4			
		438.8	338.4	9.60E4	1.30E5	1.4			
NapA	LPADMVVANPK	586.1	529.3	1.90E5	5.00E4	−3.8	38.3	7.3	−5.2
		586.1	960.6	3.70E4	8.50E3	−4.4			
		586.1	889.6	1.80E4	4.80E3	−3.8			
TorA	IAWTSNEQR	552.9	460.9	4.60E5	7.10E4	−6.5	19.3	11.3	−1.7
		552.9	543.9	3.00E5	6.30E4	−4.8			
		552.9	734.4	1.90E5	3.40E4	−5.6			

1 For each peptide, three SRM transitions were measured.

2 Intensity values were represented by integrated peak area obtained from extracted ion chromatograms.

3 Fold change of fragment ion intensities between the 2 h and 20 h samples; negative values denote decreased levels in the 20 h samples.

4 Spectral counts obtained for the same biological replicate.

5 Fold change of peptide spectral counts between the 2 h and 20 h samples; negative values denote decreased levels in the 20 h samples.

6 Peak not detected due to low peptide ion signals.

7 Fold change not determined. In this case, a large negative fold value would be expected.

We have previously shown that intracellular *C. jejuni* at late time points of infection (e. g. >18 hs) can be rendered culturable under standard microaerophilic conditions if pre-incubated under oxygen-limiting conditions (O_2_<0.2%, CO_2_ ∼6.5%) [14]. Therefore, to gain further insight into *C. jejuni*'s intracellular metabolic reprogramming, we carried out a proteomic analysis of *C. jejuni* obtained immediately after its incubation under oxygen-limiting conditions. We reasoned that by examining changes induced by this incubation step, which render it culturable, we might be able to gain further insight into the changes undergone by *C. jejuni* when located within the cells, which render it non-culturable. Bacteria obtained from infected cells 20 hs after infection and incubated for 48 hs under low oxygen conditions were scraped from plates and their proteome was examined as described above (Dataset S5). We observed significantly increased levels of enzymes involved in known *C. jejuni* anaerobic respiration pathways including FrdA, NapA, TorA, and NrfA [12] (Dataset S6). In addition, the levels of the anaerobic C4-dicarboxylate transporters DcuA and DcuB were also increased after incubation under low oxygen conditions. Strikingly, we also observed that the levels of several enzymes involved in aerobic respiration were also increased after incubation under oxygen-limiting conditions. For example, all three subunits of ubiquinol-cytochrome c reductase (PetA, PetB and PetC) were markedly increased under these conditions. These results may provide an explanation for the observation that pre-incubation of intracellular *C. jejuni* under oxygen-limiting condition renders it ready for growth under microaerophilic environment, in which oxygen acts as the main electron acceptor [14]. Furthermore, it suggests the possibility that the low oxygen environment encountered by *C. jejuni* in the gut may also prepare the bacterium for its phase outside the host, in which oxygen levels are likely much higher. Interestingly, the ability to prepare for a change in a future environment has been previously observed in *V. cholera*, in which genes required for the aquatic environment are induced within the intestinal track, where they are not needed [30].

**Figure 4:**
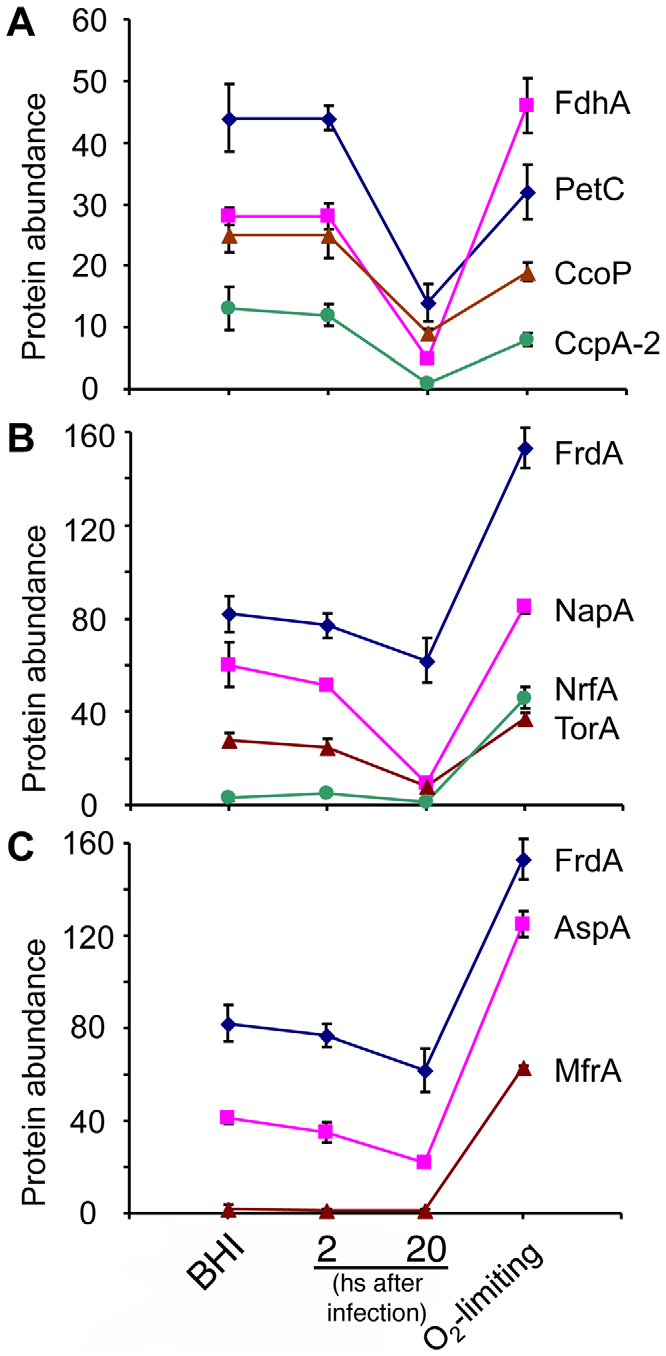
Protein abundance of respiration/electron transport chain components of *C. jejuni* in four distinct environments. Protein abundance (expressed as spectral counts) in *C. jejuni* samples obtained after growth in BHI, after 2 and 20 hs after infection, and after exposure to oxygen limiting conditions subsequently to their recovery 20 hs after infection (indicated as O_2_-limiting). **A** Protein abundance of representative enzymes involved in aerobic respirations. **B** Protein abundance of representative enzymes involved in anaerobic respirations. **C** Protein abundance of enzymes relevant to fumarate respiration. Values represent the mean ± standard error of the mean of three determinations.

To gain more insight into the potential respiratory reprogramming of *C. jejuni* within mammalian cells we specifically compared the relative abundance of key enzymes for different respiration pathways under the different conditions examined in this study. We specifically examined the levels of FdhA, PetC, CcoP and CcpA, which are involved in aerobic respiration (Figure 4A), and FrdA, NapA, NrfA, and TorA, which are central components in the respiration of fumarate, nitrate, nitrite and TMAO/DMSO, respectively (see Figure 4B) [11], [31], [32]. For quantification we used both spectral counting (Figure 4A and 4B) as well as SRM (Table 3), which yielded equivalent results. We found that the levels of aerobic respiration enzymes were markedly decreased in intracellular *C. jejuni* 20 hours after infection. A similar pattern was observed in the case of NapA and TorA. Although the levels of NrfA remained unchanged during infection, the actual protein levels were very low suggesting that this respiration pathway may not be central for the metabolism of *C. jejuni* during its intracellular state. The levels of FrdA also remained unchanged in *C. jejuni* samples 2 and 20 hs after infection. However, in contrast to NrfA, the levels of FrdA were high, suggesting that fumarate may be an important electron acceptor during *C. jejuni*'s intracellular stage. Consistent with this hypothesis, the levels of AspA, which is an aspartase that can provide fumarate for respiration [33], showed a similar pattern to the levels of FrdA (Figure 4C), which were characterized by unchanged although high levels 20 hs after infection. Furthermore, the levels of AspA, FrdA and MfrA, another fumarate reductase [11], significantly increased after incubation under low oxygen conditions (Figure 4C).

**Figure 5:**
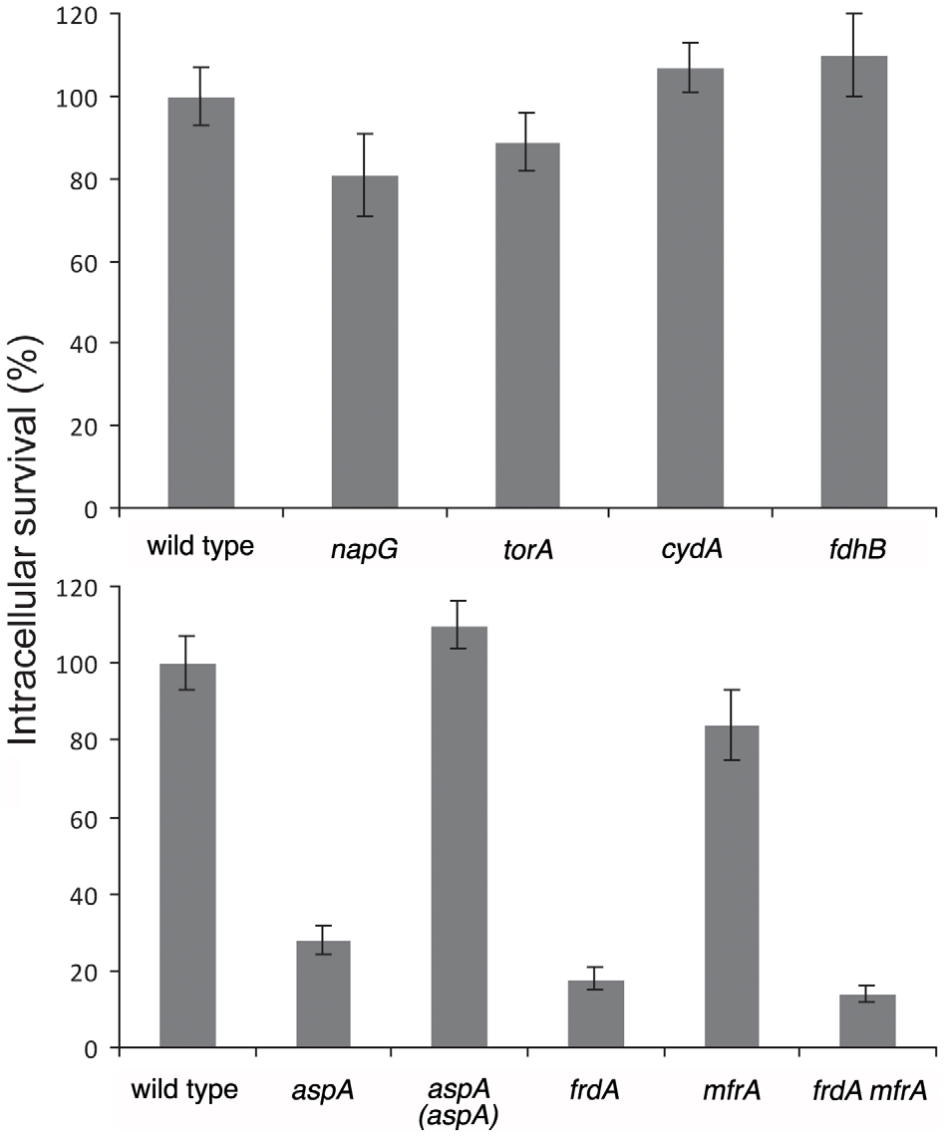
Intracellular survival of *C. jejuni* mutant strains defective in specific respiration pathways. Cultured mammalian cells were infected with the wild type *C. jejuni* 81–176 strain (WT) and the indicated mutant derivatives at a MOI of 100 for 2 hs, followed by 2 and 18 hs incubation in the presence of gentamicin. For each strain, levels of intracellular bacteria at 20 hs after infection are shown relative to the levels obtained at 2 hs after infection. The value of the ratio in wild type was set at 100%. The error bars represent the standard deviation of three independent determinations. The survival values at 20 hs of the *aspA*, *frdA*, or *frdA mfrA* double mutant strains were statistically significantly different (*P<0.05*, Student *t* test) from those of wild type.

To test the potential significance of fumarate respiration during C. jejuni intracellular survival, we constructed *C. jejuni* strains carrying mutations in *aspA* or the fumarate reductases *frdA* and/or *mfrA* and examined their viability 20 hs after infection. Since in the absence of fumarate the *C. jejuni aspA*, *frdA*, or *mfrA* mutants have been shown to be defective for invasion, these mutant strains were grown and allowed to infect cells in the presence of fumarate, which results in wild type levels of bacterial internalization [34]. The *aspA, frdA* and *frdA/mfrA* mutants showed a significant decrease in viability 20 hs after infection (Figure 5, bottom graph) indicating that respiration of fumarate is central to the metabolism of intracellular *C. jejuni*. Complementation of the *aspA* mutant restored viability to wild-type levels. In contrast, the *mfrA* mutant showed intracellular survival close to wild type. This is consistent with the extremely low levels of MfrA (barely detectable by our LC-MS approach) observed in both extracellular and intracellular *C. jejuni* preparations. These data are also consistent with previous observations indicating that *C. jejuni aspA* or *frdA* mutant strains are impaired in chicken colonization while the *mfrA* mutant is not [30] [28]. To further corroborate the hypothesis that fumarate respiration plays an important role in *C. jejuni* intracellular survival, we examined *C. jejuni* strains carrying mutations in *napG, torA, cydA*, and *fdhB* for their ability to survive within cultured mammalian cells. These genes are involved in aerobic and anaerobic respiration pathways [29] [32]. In contrast to mutants with impaired fumarate respiration, these mutant strains showed intracellular viability indistinguishable from that of the wild-type (Figure 5). Therefore, our proteomic and functional data indicate that fumarate respiration plays an important role in *C. jejuni* intracellular survival.

### Conclusions

We have shown here through a detailed proteomic study that *C. jejuni* undergoes a significant metabolic reprogramming upon internalization within mammalian cells. A salient feature of this metabolic reprogramming is a significant metabolic downshift. This metabolic downshift is qualitatively different from the one associated with *C. jejuni*'s transition to stationary phase and therefore represents a unique metabolic state presumably associated with its adaptation to the intracellular environment. It is also consistent with the observation that although *C. jejuni* survives within mammalian cells, it does not replicate [14]. It is therefore possible that the intracellular environment, presumably shielded from many innate immune defense mechanisms, may serve as a reservoir for this pathogen but not necessarily as a replication site. The proteomic profile indicates that although non-replicating, *C. jejuni* is certainly not subject to environmental stress in this intracellular environment since none of the proteins usually associated with responses to different environmental stress showed increased levels. Another salient feature of the intracellular metabolic reprogramming is a change in respiration mode, which may be responsible for its decreased culturability under microaerophilic conditions in which oxygen is the main electron acceptor. Indeed, our data suggest that *C. jejuni* may favor fumarate respiration inside mammalian cells. Consistent with our data, *C. jeuni aspA* and *frdA* mutants, which are defective for fumarate respiration, were shown to be significantly impaired for chicken colonization [30] [28], while mutations in genes essential for other respiration pathways were not *[Olson, Appl Environ Microbiol. 2008 Mar;74(5):1367–75.]*. Therefore, this study highlights the importance of metabolic reprogramming in the biology of *C. jejuni* and may help the development of novel anti microbial strategies that would target relevant metabolic pathways.

## Materials and Methods

### Bacterial strains, cell lines, and culture conditions

The *C. jejuni* 81–176 wild-type strain, its *aspA* mutant, and the complemented *aspA* mutant derivative have been previously described [34], [35]. All other mutant strains were constructed as previously described [22]. Bacteria were routinely grown on blood agar plates (tryptic soy broth agar supplemented with 5% sheep blood) or in brain heart infusion (BHI) broth at 37°C under 10% CO_2_ (microaerophilic) or low oxygen conditions (GasPak Plus, BD-Diagnostic Systems, New Jersey). All *C. jejuni* strains were stored at −80°C in BHI broth containing 30% glycerol. COS-1 (African green monkey kidney fibroblast-like cell line) cells were obtained from the American Type Culture Collection (Manassas, VA) and grown in Dulbecco's modified Eagle medium (DMEM) supplemented with 10% bovine calf serum (BCS). All cell lines were grown under an atmosphere of 5% CO_2_.

### *C. jejuni* infection of mammalian cells and isolation of intracellular bacteria

In order to obtain sufficient amounts of bacterial proteins for mass spectrometric analysis (see strategy described below), we determined that ∼5×10^7^ to 10^8^ bacteria (equivalent to ∼50–100 µg of protein yield) would be needed for proteomic analysis. To obtain this number of bacteria *C. jejuni* infections were carried out in 15-cm plates as previously described [34] with a multiplicity of infection of 1000. Infection was allowed to proceed for 2 hs in HBSS medium. Subsequently, cell monolayers were washed extensively with pre-warmed HBSS and incubated in gentamicin-containing (100 µg/mL) DMEM media for another 2 hs to kill extracellular bacteria. After gentamicin treatment, cells were washed and lysed to release intracellular bacteria. Alternatively, the culture media was replaced with fresh DMEM containing lower concentration of gentamicin (10 µg/mL) and cells were further incubated for 16 hs before releasing intracellular bacteria.

A two-step differential centrifugation strategy was developed to separate intracellular bacteria from host cell debris. COS-1 cells were lysed in a buffer containing 0.5% Triton X-100, 20 mM Tris-HCl (pH 7.6) and 150 mM NaCl. The sample was first pelleted at 300× g for 5 min to remove host cell nuclei, and then the post-nuclear supernatant was centrifuged again at 3000× g for 20 min. These conditions resulted in the recovery of most of the bacteria in the second pellet as shown by CFU assays. The resulting bacterial pellets were washed extensively with RIPA buffer to minimize host-protein contamination.

### A general proteomic strategy for profiling *C. jejuni* proteins

We carried out proteomic analysis of proteins extracted from different *C. jejuni* samples by LC-MS/MS. The experimental design is depicted in Figure 1. Briefly, intracellular *C. jejuni* were harvested from mammalian cells at 2 hs and 20 hs of infection by differential centrifugation as described above. Bacterial cell lysates were separated by 10% SDS-PAGE to pre-fractionate the bacterial proteins. Electrophoresis was stopped when the dye front reached 1.5 cm below the stacking gel. Subsequently, the gel was divided equally into three slices, each slice was subjected to in-gel protein digestion (see below), and extracted peptide samples were analyzed by LC-MS/MS for protein identification. Spectral counts associated with protein assignments were utilized to quantitatively assess their relative abundance in each sample. Triplicate LC-MS/MS runs were conducted for each sample, thereby allowing the assessment of the technical reproducibility of the measurements. Statistical analysis was performed on data obtained from 7 biological replicates. Each replicate was comprised of two samples corresponding to two bacterial populations (from 2 hs and 20 hs of infection). Since each bacterial sample was divided into three gel fractions and each fraction was run in triplicate, in total 126 LC-MS/MS experiments were carried out to obtain the entire data set. Protein and peptide assignments from different gel fractions were pooled within the same bacterial sample. All mass spectrometric data have been deposited at the public data base PRIDE hosted by EMBL-EBI (http://www.ebi.ac.uk/pride/)

### In-gel digestion of protein samples

Gel slices were excised into small (∼1×1 mm) cubes and transferred into sample tubes. Protein disulfide bonds were reduced with 10 mM DTT in 100 mM NH_4_HCO_3_ at 56°C for 30 min and subsequently alkylated with 55 mM iodoacetamide (IAM) in 100 mM NH_4_HCO_3_ at room temperature for 20 min (in complete darkness). Upon alkylation, the gel samples were destained with 50% acetonitrile (ACN) in 50 mM NH_4_HCO_3_ and dehydrated by addition of neat ACN. After removal of the destaining buffer and ACN, the samples were subjected to in-gel digestion. The digestion buffer contained 10 ng/µL trypsin in 50 mM NH_4_HCO_3_ and the enzymatic reaction was allowed to proceed overnight at 37°C. The resulting tryptic peptides were extracted from the gel matrix by equilibrating the samples with 50% ACN and 5% formic acid (FA). Finally the extracted peptides were vacuum dried prior to LC-MS/MS analysis.

### Nanoflow LC-MS/MS analysis

Nanoflow reverse-phase LC separation was carried out on a Proxeon EASY-nLC System (Thermo Scientific). The capillary column (75 µm×150 mm, PICOFRIT, New Objective, Woburn, MA) was packed in-house. Briefly, a methanol slurry containing 5 µm, 100 Å Magic C18AQ silica-based particles (Microm BioResources Inc., Auburn, CA) was forced to pass through an empty capillary (with a frit in the end) using a pressurized device. Tryptic peptides were dissolved in HPLC-grade water and ∼200 ng of samples were loaded onto the analytical column in a single LC-MS/MS experiment. The mobile phase was comprised of solvent A (97% H_2_O, 3% ACN, and 0.1% FA)) and solvent B (100% ACN and 0.1% FA). The LC separation was carried out with the following gradient: solvent B was started at 7% for 3 min, and then raised to 35% in 40 min; subsequently, solvent B was rapidly increased to 90% in 2 min and maintained for 10 min before 100% solvent A was used for column equilibration. Peptides eluted from the capillary column were electrosprayed directly onto a linear ion trap mass spectrometer (LTQ Velos, ThermoElectron, San Jose, CA) for MS/MS analysis. A data-dependent mode was enabled for peptide fragmentation. One full MS scan was followed by fragmentation of the top 10 most intense ions by collision-induced dissociation (CID). Dynamic exclusion (with a duration of 6 seconds) was enabled to preclude repeated analyses of the same precursor ion. Both pre-column and analytical column were washed intensively between different samples to remove any carryover from previous runs.

### Protein identifications and quantitative analysis of their relative abundance

Peptides and proteins were assigned by a database search method. Specifically, MS/MS scans were processed with commercial software (Bioworks Browser, ThermoElectron, San Jose, CA) to generate DTA files. Then those files were searched with MASCOT (Matrix Science Ltd., London, UK) against a *C. jejuni* 81–176 protein database. The resulting peptide and protein assignments were filtered to achieve a peptide false discovery rate (FDR) of <1% and a protein FDR of <3% using the target-decoy method (e. g. reverse database search). Protein relative abundance between different samples was assessed using a spectral counting method. Spectral counts represent the total number of repeated identification of peptides for a given protein during the entire analysis and provides a semiquantitative measurement of protein abundance. Raw spectral counts were normalized against constant protein signals (between 2 h and 20 h samples). Eight highly abundant *C.jejuni* proteins (GroEL, AcnB, flagellin, pyruvate ferredoxin, RpoB, RpoC, Tpx, methyl-accepting chemotaxis protein/CJJ81176_1205) having little change in expression level during the course of infection were chosen to calculate the final normalization factor. A protein fold change (or ratio) between paired samples was calculated by dividing the average spectral counts of triplicate measurements. Then the averaged value of ratios for 7 biological replicates was calculated for each detected protein. A ratio (20 h over 2 h samples) greater or smaller than 1 indicates that levels of a given protein in 20 h samples were higher or lower, respectively, than those at 2 h after infection. Paired Student's t test was performed on data from 7 biological replicates. For the purpose of our analysis, differences between the average fold change of a given protein 2 h and 20 h after infection of >2 and a *p*-value<0.05 were considered significant. Among those proteins that met our criteria, a subset of metabolic enzymes of particular interest to this study were further examined by selected reaction monitoring (SRM).

### *C. jejuni* infection of cultured mammalian cells

*C. jejuni* infection of cultured mammalian cells was also carried out as previously described [14] with an MOI of 100. In all cases, the *C. jejuni aspA, frdA* or *frdA mfrA* double mutant strains were grown in the presence of fumarate and fumarate was added during the initial infection of cultured mammalian cells. After 2 hs of infection, cells were washed extensively with HBSS followed by the addition of pre-warmed DMEM containing 100 µg/mL gentamicin for 2 hs to kill extracellular bacteria. Cells were lysed in a buffer containing 0.5% Triton X-100, 20 mM Tris-HCl (pH 7.6), and 150 mM NaCl, and the number of intracellular bacteria was assessed by CFU assays. Alternatively, infected cells were washed extensively and incubated overnight with DMEM containing 10 µg/ml of gentamicin. Cells were then lysed as described above and the released intracellular bacteria were plated and incubated under low-oxygen conditions for 2 days before further incubation under microaerophilic conditions as previously described [14]. The ability of *C. jejuni* to survive intracellularly was assessed as a percentage of bacteria recovered after 20 hs incubation relative to those recovered 2 hours after infection.

## Supporting Information

Dataset S1 Spectral counts of *C. jejuni* proteins detected by LC-MS/MS.
(XLS)

Dataset S2 *C. jejuni* proteins whose levels exhibit significant changes between the 2 h and 20 h samples.
(XLS)

Dataset S3 *C. jejuni* proteins detected in in-vitro grown or obtained from infected cells 2 hs after infection.
(XLS)

Dataset S4 Spectral counts of *C. jejuni* proteins detected by LC-MS/MS after exposure to infection medium.
(XLS)

Dataset S5 *C. jejuni* proteins detected 20 hs after infection or after incubation under oxygen -limiting conditions.
(XLS)

Dataset S6 *C. jejuni* proteins whose levels exhibit significant changes between 20 h after infection and after incubation under low oxygen conditions.
(XLS)
